# Bird to Human Transmission Biases and Vaccine Escape Mutants in H5N1 Infections

**DOI:** 10.1371/journal.pone.0100754

**Published:** 2014-07-02

**Authors:** Kshitij Wagh, Aatish Bhatia, Benjamin D. Greenbaum, Gyan Bhanot

**Affiliations:** 1 Department of Physics, Rutgers the State University of New Jersey, Piscataway, New Jersey, United States of America; 2 The Cancer Institute of New Jersey, New Brunswick, New Jersey, United States of America; 3 Departments of Medicine, Division of Hematology and Medical Oncology, and Pathology; and the Tisch Cancer Institute, Icahn School of Medicine at Mount Sinai, New York, New York, United States of America; 4 The Simons Center for Systems Biology, Institute for Advanced Study, Princeton, New Jersey, United States of America; 5 Department of Molecular Biology & Biochemistry, Rutgers University, Piscataway, New Jersey, United States of America; 6 BioMaPS Institute, Rutgers University, Piscataway, New Jersey, United States of America; Virginia Polytechnic Institute and State University, United States of America

## Abstract

**Background:**

The avian influenza A H5N1 virus occasionally infects humans, with high mortality rates. Although all current human infections are from avian-to-human transmission, it has been shown that H5N1 can be evolved to transmit between mammals, and is therefore a pandemic threat. For H5N1 surveillance, it is of interest to identify the avian isolates most likely to infect humans. In this study, we develop a method to identify mutations significantly associated with avian to human transmission.

**Method:**

Using protein sequences for the surface glycoprotein hemagglutinin from avian and human H5N1 isolates in China, Egypt, and Indonesia from the years 1996–2011, we used Principle Component Analysis and a Maximum Likelihood Multinomial method to identify mutations associated with avian to human transmission. In each geographic region, transmission bias residues were identified using two signatures: a) significantly different amino-acid frequencies in human isolates compared to avian isolates from the same year, and b) significantly low probability of neutral evolution of the human isolates from the avian viral pool of the previous year.

**Results:**

In each geographic region, we find specific transmission bias mutations associated with human infections. These mutations are located in antigenic regions and receptor binding, glycosylation and polybasic cleavage sites of HA. We show that human isolates derive from a limited, subset of the avian pool characterized by geography specific mutations. In Egypt, two of three PCA clusters have very few human isolates but are highly enriched in mutations associated with a vaccine escape mutant H5N1 avian sub-clade that is known to be resistant to the Mexican H5N2 vaccine Furthermore, at these transmission bias associated residues, the mutations characteristic of these two clusters are distinct from those associated with the cluster enriched in human isolates, suggesting that vaccine resistant avian strains are unable to infect humans. Our results are relevant for surveillance and vaccination strategies for human H5N1 infections.

## Introduction

The H5N1 Influenza A avian virus is an existing pandemic threat [1]–[4]. Although human H5N1 infections occur rarely, such infections are usually accompanied by severe respiratory complications with high morbidity, and a mortality rate approaching 60% [5]–[6]. Infections in humans occur almost exclusively from direct human contact with infected wild birds or poultry. Currently, the poor human-to-human transmission efficiency of circulating H5N1 strains [7] limits their pandemic potential. However, this poor transmission can be overcome by evolution of H5N1 in mammalian hosts [8]. Laboratory studies of experimentally evolved H5N1 strains show that current strains can transmit efficiently between mammals (ferrets) with only 4–5 substitutions at specific residues in hemagglutinin (HA) and polymerase basic 2 (PB2) proteins [9], [10]. Given the high mortality rate of human infections from currently circulating avian strains, there is an urgent need to identify which avian H5N1 strains are most likely to infect humans.

Vaccination of poultry has been undertaken to control H5N1 infections in several countries such as China, Egypt, Indonesia and Viet Nam, where H5N1 is endemic [11]. Several inactivated reassortant H5N1, H5N2 and H5N3 vaccines as well as vector vaccines have been developed and used. The vaccinations have shown limited efficacy and vaccine resistance has been observed [12]. Therefore it is imperative that vaccines be redesigned for efficacy against the prevalent strains. In Egypt, for example, the attenuated H5N1 strain vaccines predominantly used in domestic poultry have changed from using a 1996 strain (until 2008), to 2006 strains (2009–2012), to a 2009 strain (2012 onwards). However, as of 2011, the vaccine predominantly used in commercial poultry farms is a Mexican 1994 H5N2 strain vaccine [12]. It is thus important to understand how the H5N1 virus is evolving under vaccination induced selection pressure. This issue has important implications for surveillance of the avian viral pool, intelligent vaccine design, and, most significantly, identifying avian strains likely to jump into human hosts.

Since almost all human H5N1 infections so far were transmitted from avian hosts, investigating and understanding any biases in such inter-host-species transmission is important. Any observed signature of biased transmission from birds to humans could represent enhanced/diminished efficiency due to specific functional mutations possessed by certain H5N1 strains which cause them to infect human hosts with greater efficiency. Selection in H5N1 viruses infecting humans has been studied previously [13]–[15] using differences in the rates of synonymous and non-synonymous mutations in human isolates as the characteristic signature for selection. However, because H5N1 is transmitted from birds to humans, with little to no known transmission between human hosts, such an analysis cannot distinguish between selection pressures on H5N1 from avian infections versus human transmissions. In other words, analysis of H5N1 viruses solely from human subjects cannot identify which mutations in the avian H5N1 pool are important for transmission from birds to humans, compared to those which only have a selective advantage in birds. In this paper, we develop a simple strategy to identify and interpret such H5N1 transmission bias mutations which are important in infections from avian to human hosts.

**Figure 1:**
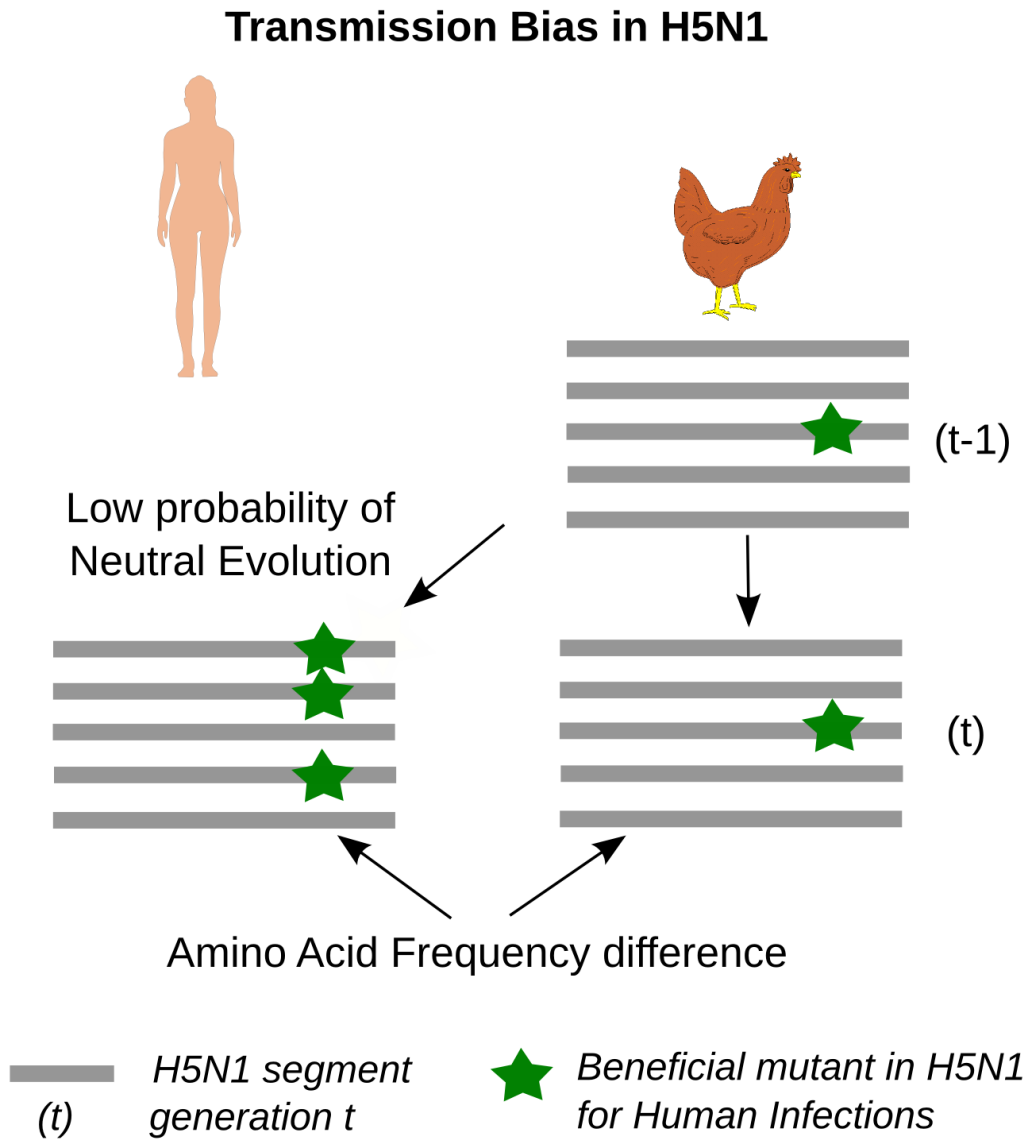
Methods to detect transmission Bias of H5N1 strains from birds to humans. A hypothetical scenario of a mutation under positive selection for human infections, but selectively neutral in avian infections, will result in a transmission bias of H5N1 strains from birds to humans. Such a residue would show: a) Significant increase in the frequency of residues in human isolates compared to their frequency in the avian viral pool; b) Low probability that the current human isolates are derived from neutral evolution of the avian viral pool of the previous year. We use these two tests to identify transmission-bias in human infections.

A mutation conferring higher infectivity in human infections but neutral in avian infections would be over-represented in human isolates, but not in avian isolates (Figure 1). Such mutations would have two characteristic signatures: a) a significant difference in amino-acid frequencies in human isolates compared to avian isolates from the same year and b) a significantly low probability of neutral evolution of the human isolates from the avian viral pool of the previous year. We applied these criteria to protein sequences of H5N1 avian and human isolates from 1996–2011 collected in China, Egypt and Southeast Asia. We analyzed strains from each geographic region separately, and corrected for population sub-structure within each region using Principal Component Analysis. For this study, we focused on the protein hemagglutinin because of its key role in host-cell receptor binding and antigenicity [16], [17]. For each geographic region, on an annual resolution, our methods identified residues that show biased transmission from birds to humans. We find that, in each geographic region, strains infecting humans originate from a subset of the avian viral pool characterized by specific mutations at identified residues (“transmission bias mutations/residues”). The identified transmission bias residues are in immunologically and functionally relevant regions of HA, such as the epitope regions, the receptor-binding site, the polybasic cleavage site and the trans-membrane site. In Egypt, at transmission bias residues conserved in human isolates, we find that human isolates are significantly different from avian isolates resistant to Mexican H5N2 vaccine. This raises the possibility that these vaccine-resistant avian strains may be unlikely to infect humans. This result is important because it suggests that appropriate vaccine pressure in birds may drive the H5N1 virus away from being able to infect humans. However, further experimental and environmental studies are needed to verify this possibility.

## Results

### Human H5N1 isolates derive from a subset of avian viruses with geography-specific epitope profiles

**Figure 2:**
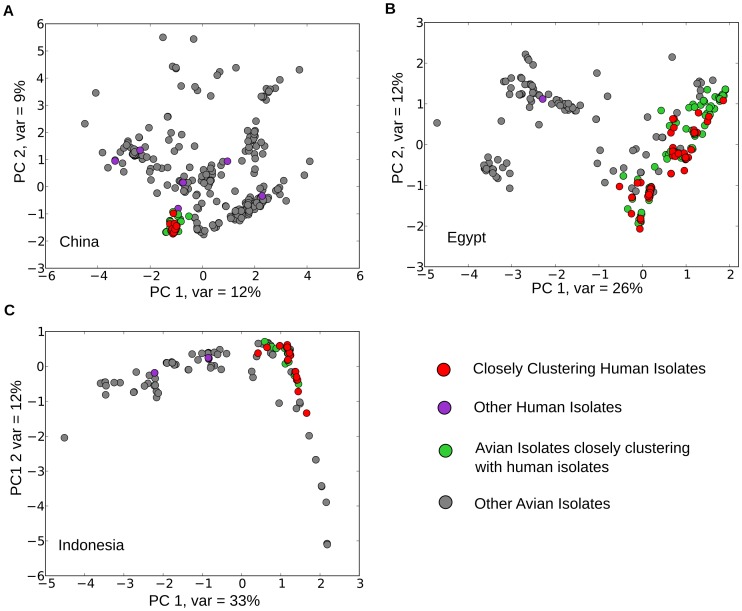
Population substructure and transmission bias in H5N1 strains. The figure shows the first two principal components from PCA of HA amino acid sequences from avian and human isolates from China (A), Egypt (B) and Indonesia (C). Closely clustering human and avian isolates, in each region, were identified using an algorithm which clusters strains by using a distance cutoff in principal component space (Methods).

We analyzed 1209 HA sequences of H5N1 isolates from avian (n = 1056) and human hosts (n = 153) from China, Indonesia and Egypt, collected from 1996–2011. Principal Component Analysis (PCA) was used to study population structure (Methods). PCA plots for HA sequences from each geographic region are shown in Figure 2. In each geographic region, human isolates cluster with subsets of avian isolates, suggesting a transmission bias in H5N1 infections from avian hosts to humans. To characterize the subsets of avian isolates most likely to infect humans, we identified clusters of closely related human and avian isolates, using a distance cutoff in PCA space (Figure S1, Methods). The identified clusters consist of most of the human isolates in each region: 30 out of 36 in China, 70 out of 71 in Egypt, and 46 out of 50 in Indonesia (details on cluster membership in Tables S1 A–C).

We used two signatures to identify transmission bias residues in avian to human infections: a) The residues should have a significant difference in amino-acid frequency in human isolates compared to avian isolates from the same year and b) The residues in human isolates should have a significantly low probability to derive from neutrally evolved avian viral isolates from the avian viral pool of the previous year. To evolve the avian pool neutrally from one year to the next, we adapted the method of Pan and Deem [18]. The expected frequencies of amino acids at a given locus in a given year were obtained by neutrally evolving the observed amino acid frequencies at this locus in the previous year, using an influenza-specific amino acid transition probability matrix calculated by Dang et al. [19]. Using the avian isolate frequencies (either actual or expected under neutral evolution from the previous year) as a-priori human isolate amino-acid frequencies, we used a multinomial analysis to estimate the probabilities of the observed human isolate amino-acid frequencies. A Jackknife test was used to determine significance cutoffs. The most significant residues thus identified are listed in Table S2 by year and geography. Amino acid frequencies for these residues are in Table 1 and Table S3.

**Table 1:** Transmission bias mutations that are conserved or at high frequency in closely clustering human and avian isolates.

Position (H5 numbering)	Conserved/High Frequency amino acid in human isolates in cluster	Average Frequency of conserved/high frequency Amino Acid (%) in			Year of first report of conserved amino acid			
		Human isolates not in cluster	Avian isolates in cluster	Avian isolates not in cluster	Avian Isolates	Human Isolates	Other Amino Acids at residue	Region of HA^a^
Egypt	(n = 70)^b^	(n = 1)	(n = 195)	(n = 140)	(first reported in 2005)^c^	(first reported in 2006)		
**74**	P	0	100	32.9	2005	2006	S	∼^d^Epi eE
**97**	D (98.6)^f^	0	99	29.3	2005	2006	N, E, del	-
**110**	H	0	100	33.6	2005	2006	R, G	Epi A
**123**	S	0	99.5	37.9	2005	2006	P, L	∼Epi A, ∼RBS^g^
**141**	S (97.1)	0	94.9	23.6	2005	2006	P, L	Epi B
**144**	F	0	100	31.4	2005	2006	Y, C	Epi B
**165**	N	0	100	36.4	2005	2006	H	GS^h^
**226**	M (97.1)	0	89.2	22.9	2005	2006	V, I	Epi D

Indonesia	(n = 42)	(n = 4)	(n = 50)	(n = 128)	(first reported in 2003)	(first reported in 2005)		
**86**	T	0	90	19.5	2005	2005	A, N	Epi D
**94**	S	0	100	36.7	2004	2005	N, D, M, del, K	∼Epi B
**140**	S	0	100	32.0	2005	2005	K, T, Q, R, D, N, del	Epi B
**200**	I	0	100	25.0	2003	2005	V, del	Epi D
**325**	S	0	98	19.5	2005	2005	R, A, G	PBS^i^

China	(n = 30)	(n = 6)	(n = 66)	(n = 394)	(first reported in 1996)	(first case in 2005)^j^		
**140**	T	0	100	17.5	2004	2005	R, K, S, N, E, M, A, V	Epi B
**174**	I (86.7)	33.3	97	24.4	1999	2005	V	Epi B, ∼RBS
**181**	S	16.7	95.5	23.4	2004	2005	P, F	Epi B
**322**	L	16.7	100	21.6	2005	2005	Q, P, del, H, K, R, S	∼∼∼^k^PBS

Conserved residues and residues with high-frequency amino acids in closely clustering human isolates which have significantly low probability to neutrally evolve from and significant differences in amino-acid frequencies from the entire avian viral pool of each geographical region and year. Frequencies for other significant mutations are in Table S3.

a Information on the function of residues is taken from Duvvuri et al. [11].

b The numbers in the rows next to each country's names indicate the number of samples in each class.

c The years of first report in each country as per the information available in the NCBI Influenza Virus Resource database.

d “∼” indicates adjacent residue.

e Epitope.

f Frequency in percent.

g Receptor-binding site.

h Glycosylation site.

i Polybasic cleavage site.

j The first human isolate of H5N1 reported was in 2003, but there was contiguous reporting of human isolates only since 2005.

k 3 residues upstream.

**Figure 3:**
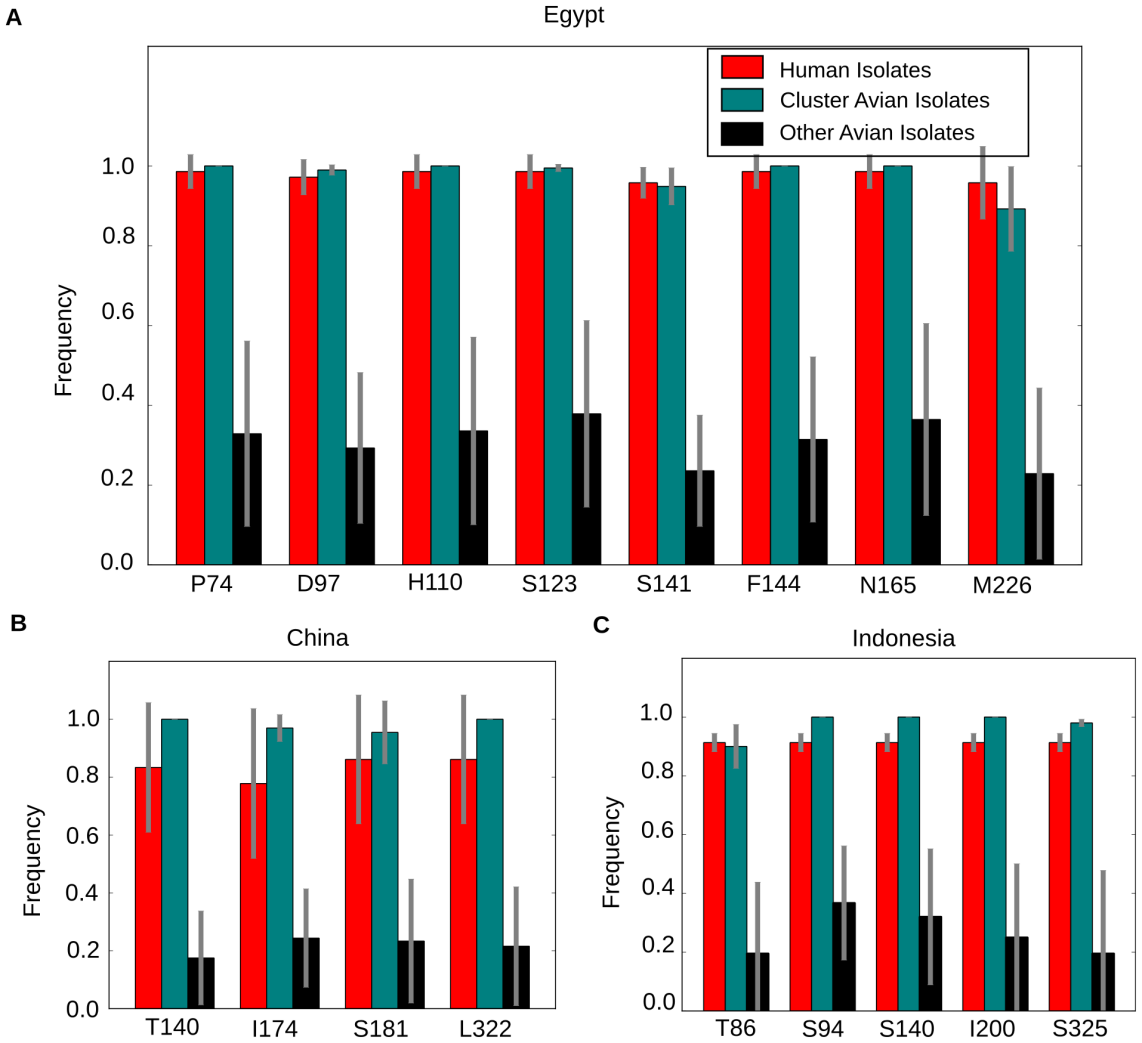
Average annual frequency of significant HA mutations responsible for geography specific transmission bias of H5N1. Average annual frequencies of the major amino-acid at significant residues (Table S1) for human isolates (red), avian isolates which cluster with human isolates (teal), and other avian isolates (black) from Egypt (A), China (B) and Indonesia (C). The grey bars represent two standard deviation variation in the observed annual frequency.

Several of these residues have one high frequency (>80%) amino acid in the human isolates in each region (Figure 3 and Table 1). Most of the human isolates in each geographical region cluster together (80–99%) (Figure 2) and these amino acids are virtually conserved in these closely clustering human isolates. These amino acids are also almost conserved (frequencies >89%) in closely clustering avian isolates, but have low to intermediate frequencies (18–38%) in other avian isolates (see Methods for the definition of “closely clustering” isolates). As indicated by the similarity of amino acid frequencies at all transmission bias residues between human and closely clustering isolates (Table 1), we found that at these residues the strains infecting humans are most likely to have evolved neutrally from the closely clustering avian isolates (Table S2, columns E, F). These results, taken together, suggest that for each geographic region, human infections are significantly more likely to arise from an identifiable subset of avian isolates, characterized by specific amino acids at the residues identified in Table 1, rather than from the entire avian viral pool.

Many of the loci associated with the transmission bias are located in or near functional regions of HA, such as the epitope regions (corresponding to epitopes B, D and E in H3 HA), the receptor-binding site, the polybasic cleavage site, and the trans-membrane region (Table S2). Given that some of the transmission bias residues are in epitopes, avian strains most likely to infect humans could have distinct epitope profiles from other avian strains. The mapping of these residues onto the protein structure of H5N1 HA [17] shows that most of these residues are in the head region of the HA protein structure (Figure S2). We also find that all except one of the high frequency residues identified in Table 1 arose in the avian viral pool of the region in either the same or the previous year, as first reported in human infections, which further suggests their relevance to human infections.

### In Egypt, H5N1 strains resistant to Mexican H5N2 vaccine are less likely to infect humans

Avian strains in Egypt have undergone diversification in response to vaccine induced selection pressure in poultry [20], [21]. These antigenically drifted avian isolates are now classified as a variant group within the sub-clade 2.2.1 (group I in [21]). The mutations characteristic of these isolates are S-74, N-97, R-110, P-123, G-140, P-141, Y-144, K/E-162, H-165, E-184, and V-226 [19]. Our results identified all but two (G-140 and E-184) of the residues characterizing this vaccine resistant avian H5N1 group (Table 1 and Table S2). However, *the mutations characterizing human isolates were distinct from the mutations characteristic of the escape mutant group*. Specifically, residues 74, 97, 110, 123, 144, and 165 have virtually conserved amino-acids in closely clustering human and avian isolates, which are different from those characterizing the variant group of avian isolates. Since 70 out of 71 human isolates from our Egypt dataset cluster closely, this finding suggests that the variant avian strains of sub-clade 2.2.1 from vaccinated birds are unable to infect humans.

**Figure 4:**
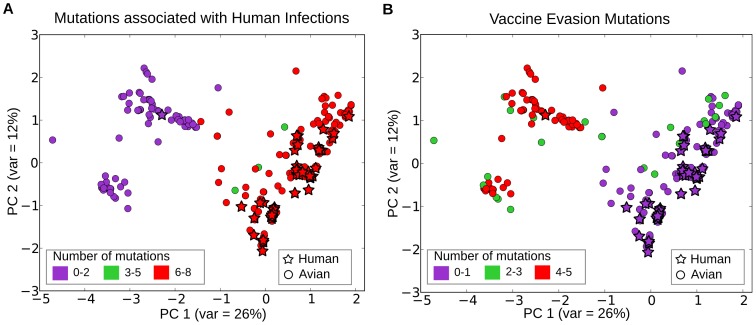
Transmission bias mutations compared to vaccine evasion mutations in Egypt. PCA plots distinguish the frequencies of transmission bias loci (A) and vaccine evasion mutations (B). In (A) transmission bias mutations P-74, D-97, H-110, S-123, S-141, F-144, N-165 and M-226 (total = 8) have a high frequency in human isolates and closely clustering avian isolates but not in other avian isolates. In (B), vaccine-evasion mutations S-74, G-140, P-141, Y-144 and K-162 (total = 5) from Cattoli et al [12], which are responsible for resistance to the Mexican H5N2 vaccine strain commonly used in commercial poultry farms in Egypt are overrepresented in a different subcluster compared to the transmission bias mutations (A).

A serological study using reverse genetically designed viruses carrying the above mentioned variant group specific mutations showed that the mutations S-74, G-140, P-141, Y-144 and K-162 are involved in escape from neutralization due to Mexican H5N2 vaccine induced antibodies in chickens [12]. A comparative PCA analysis of the H5N1 isolates from Egypt carrying the high frequency transmission bias mutations versus the vaccine evasion mutations (Figure 4A–B) found that the closely-clustering human and avian isolates carry 0–1 out of the 5 vaccine evasion mutations, whereas the more divergent avian isolates carry 3–5 mutations. We also find that the cluster of escape mutant avian isolates is distinct from the cluster containing human isolates, and carries 0–1 of the 8 high frequency transmission bias mutations (P-74, D-97, H110, S-123, S-141, F-144, N-165, and M-226). These results suggest that the mutations involved in vaccine evasion, at least in this instance, have resulted in inefficient transmission of avian H5N1 strains to humans. Thus, the potential of avian H5N1 strains to infect humans seems to have been effectively neutralized by the use of the Mexican-derived H5N2 vaccine on poultry in Egypt.

### Residues associated with human H5N1 isolates after correcting for biased transmission

We investigated the possibility of additional residues associated with human infections after correcting for the transmission biases described above. Such residues should display a) significant amino acid frequency differences between human isolates *and the subset of closely clustering avian isolates*, and b) significantly low probabilities of having evolved neutrally *from the subset of closely clustering avian isolates of the previous year*. The residues that have these properties are listed in Table S4 for each geographic region and year. The identified residues are in the epitope D region of the H3 hemagglutinin (residues 184–186 in isolates from Indonesia), and near the trans-membrane site (residue 513 in isolates from Egypt). The T513I mutation in Egypt arose in 2006, and for the years 2007 and 2008 showed enrichment in human isolates (8 out of 19 and 3 out of 7 human isolates respectively) as compared with the closest avian isolates (frequencies 0.07–0.15), but not for the year 2009 (Figure S3A). The mutation N-184 arose in 2005 in isolates circulating in Indonesia, and showed enrichment in the reported human isolates in 2007 (5 out of 5) over the avian isolates (frequency <20%) (Figure S3B). The mutations E-185 and E-186 are in close linkage with the N-184 mutation and show similar enrichment in human isolates as compared with avian isolates from Indonesia from the year 2007 (data not shown).

## Discussion

Using HA sequence data, we identified a pronounced population substructure in the H5N1 strains in each geographical region studied, with human isolates clustering together with a subset of avian isolates (Figure 2). To identify the residues that could be important for avian to human transmission of H5N1, we designed a novel analysis based on comparison of human and avian isolates using two complimentary measures (Figure 1). This analysis of avian and human isolates in each geographic region identified specific mutations characterizing subsets of avian isolates with increased potential for infecting humans (Table 1, Table S2). These residues are in or near the epitope regions, the receptor binding site, and the polybasic cleavage site of the HA proteins. They have high frequencies for specific amino-acids at specific loci in human and closely clustering avian isolates and a significantly lower frequency in other avian isolates (Table 1, Figure 3). This suggests that not all avian strains can efficiently infect humans. Instead, only an identifiable subset, with specific amino acids at identified residues, can do so. The amino acids at these identified residues seem to be important for the H5N1 viruses to infect humans in specific geographic regions.

A possible biological reasons for the enrichment of the identified mutations in human isolates relative to avian isolates might be that they are involved in efficient binding to receptors on human epithelial cells and/or in evasion of neutralization by the human immune response. It is known from the study of Yamada et al. [17] that HA from human isolates has the ability to bind to cells with both the avian-type (α2,3) and human-type (α2,6) sialic acid receptors, whereas HA from avian isolates can bind only to the avian type sialic acid receptors. Watanabe et al. [22] have studied mutations responsible for receptor binding properties of human infecting H5N1 isolates circulating in Egypt and found that isolates with increased affinity for human-type sialic acid binding also retained binding to the avian-type sialic acid receptors. They showed that mutations at residue 192 and at residues 129 in combination with 151 (also identified in Table 1) enhanced the binding to the human-type sialic acid receptors, while still retaining binding to avian-type sialic acid receptors.

However, we note that Watanabe et al. [22] also showed that an older reference avian strain (without the mutations at 129,151 and 192), but with some of the amino acids identified in Table 1, does not bind efficiently to human-type sialic acid receptors. In addition, they showed that reverse-genetically designed isolates with specific mutations at 129, 151 and 192, in the background of the reference strain, increased the virulence of H5N1 in mice as compared to the original reference avian strain. These results suggest that whereas the identified mutations in Table 1 may not be directly responsible for increased human-type sialic acid receptor binding or increased virulence, they may be involved in complex (possibly background dependent) interactions that enhance infectivity in humans. Given that the mutations identified in Table 1 are almost conserved within human isolates and closely clustering avian isolates, but are at low frequencies in the other avian isolates, and that these mutations were present in the isolates in the study by Watanabe et al., these mutations may be a pre-requisite for higher human-type receptor binding and/or higher virulence.

Another possible reason for enhanced/diminished transmission of some H5N1 strains from birds to humans could be vaccine-induced diversification of avian viruses. In Egypt, the identified residues conserved in the closely clustering human and avian isolates have also been shown to be involved in vaccine evasion [12]. Cattoli et al. studied the effect of Mexican H5N2 strain induced antibodies in chickens on a divergent clade of avian H5N1 isolates from commercial poultry farms in Egypt, where the Mexican H5N2 vaccines are used extensively. Using reverse genetics and serological studies, they found the mutations at residues 74, 140, 141, 144 and 162 to be important for the vaccine-resistance of the divergent clade of H5N1 isolates. Our results identified all the above residues, except residue 140. We find that the residues at 74, 141 and 144 are almost conserved in human isolates and are different from the ones involved in vaccine-resistance (Figures 3, 4). The mutual exclusivity of the presence of vaccine-evading mutations and the mutations enriched in human isolates (Figure 4A–B) in H5N1 strains suggests that during acquisition of vaccine-evasion, the divergent avian strains lose the ability to infect humans. China and Indonesia have also used vaccines for H5N1 control [11], and it would be interesting to investigate whether the mutations associated with human infections also provide resistance to vaccines in poultry.

The reduced efficiency of vaccine evading avian H5N1 strains to infect humans could arise due to a) low viral loads in vaccinated poultry [23] leading to reduced transmission to humans; b) the escape mutant virus being a poor transmitter in general compared to the wild-type strains; or c) vaccine induced molecular changes making the mutant strains transmit inefficiently to humans. Immune escape of influenza viruses is intimately connected with receptor binding [24], [25]. Thus, it is possible that the vaccine evasion in avian H5N1 strains could be accompanied by changes in receptor binding properties leading to a reduced infectivity in humans. In any event, our results show that vaccination with the Mexican H5N2 strain evolved the virus away from human infectivity. Cattoli et al. showed that avian reference strain with human-specific mutations can be neutralized by antibodies induced in chickens by vaccination with Mexican H5N2 [12]. Thus, although the intensive use of Mexican H5N2 vaccine has led to the development of vaccine-resistant avian H5N1 isolates, this vaccine could prove beneficial to control human infections in Egypt. To our knowledge, this is the first observation that selection pressure from some types of vaccination of poultry may be driving H5N1 away from being able to infect humans. Our analysis suggests that appropriate vaccination of poultry designed to be effective against specific epitopes enriched in human isolates may significantly mitigate the risk of human infections. However, several environmental effects, which cannot be adequately assessed from the literature, need to be taken into account. For instance, one needs to understand the fractions of human infections from backyard versus commercial poultry, and the degree to which poultry infected with vaccine escape mutant viruses came in contact with humans at all. Laboratory studies using viral epitopes identified in this paper would be the simplest way to prove or disprove our claims.

After correcting for transmission bias of H5N1 isolates from the avian viral pool to humans, we found that certain residues have a high frequency in human isolates compared to the closely clustering avian isolates. These residues may increase the likelihood of human infectivity in the particular genetic background of avian H5N1 strains that are more likely to infect humans (Table S4, Figure S3). In particular, the residues identified to have this property in Indonesian isolates are in the region corresponding to epitope D of H3 HA. The residue 513, which was identified in Egypt, lies close to the trans-membrane site of HA. Intriguingly, these residues do not have significant scores when human isolates are compared with all the avian isolates. The amino-acid frequencies at these sites in the human isolates are not significantly different from those in the entire avian pool, but differ only from the closely clustering avian isolates. This suggests the rather unusual possibility that loss of the wild type alleles at these mutations could enhance human infectivity in the genetic background of avian isolates that closely cluster with the human isolates.

In summary, in each geographic region, only certain identifiable subgroups of avian H5N1 isolates seem able to infect humans, and selection pressure from vaccination has created escape mutants that are unable to infect humans. Experimental investigation of these results would provide additional insights into the biological mechanisms underlying enhanced human infectivity of certain H5N1 strains as well as on how vaccination pressure affects the ability of H5N1 avian viruses to infect humans.

## Materials and Methods

### Sequence data

Aligned amino acid and nucleotide sequences for hemagglutinin of H5N1 isolates were downloaded from the NCBI Influenza Virus Resource database (http://www.ncbi.nlm.nih.gov/genomes/FLU/Database/nph-select.cgi), on August 8, 2012 (Egyptian isolates) and October 18, 2012 (Asian isolates). Alignment was performed using the program MUSCLE using default parameters. Identical strains were removed using both the web resource's option and additional programming (to account for identity up to missing residues) with unique human isolates preferably retained from a set of identical isolates. Host, region, and year information for all isolates wwas also downloaded from the above website. The resulting dataset comprised 1209 (153 human, 1056 avian) isolates in Egypt, China, and Indonesia from years 1996–2011. Human isolates for all geographical regions combined were from years 2005–2010.

### Principal Component Analysis (PCA)

We performed PCA on hemagglutinin amino acid and nucleotide sequence data for isolates from both avian and human hosts from each region to understand population structure. Both amino acid and nucleotide sequences in the dataset had sites with more than two variants. To encode these amino acids or nucleotides into numerical values, we used the following prescription. Amino acids at each residue were assigned values 0,1,2,…,19, with the most common variant assigned to 0, the next frequent 1, and so on. In all of the isolates in each geographic region, we excluded residues with a missing amino acid, which would indicate a deletion or missing sequence. The numerical data for each residue was normalized by subtracting the mean. However, we did not divide the result by the standard deviation to ensure that the more variant sites carry higher weight in the PCA analysis. The PCA analysis was done using the module for Singular Value Decomposition in SciPy [26].

### Identifying the avian strains that closely cluster with human strains using a distance cutoff in PCA space

PCA on H5N1 isolates from avian and human hosts in each geographic region revealed that isolates from each region exhibited signs of population substructure (Results). To understand this population sub-structure and its relevance to transmission bias, we constructed sub-clusters in PCA space by first clustering human isolates that were close to one other, and then clustering avian isolates that were close to these human isolates. More specifically, we first retained only those PCA components that accounted for >4% variance. We then constructed clusters of human isolates that fell within a distance corresponding to 4% variation of the total variation in each local region. We found that by using this distance cutoff, almost all (>80%) of the human isolates in each region clustered together.

We used the following algorithm for clustering human isolates. Initially all human isolates were placed in the un-clustered list. Because each human isolate belongs to a cluster (albeit of size one), we chose a random isolate to seed the first cluster and removed it from the un-clustered list of isolates. In the next step, all isolates within the distance cutoff from this initial isolate were included in the cluster, and removed from the list of un-clustered isolates. If the cluster size was greater than one, then new un-clustered isolates were added to this cluster if they were closer than the distance cutoff to at least one of the cluster isolates. This step was iterated until there were no more isolates in the un-clustered list that were within the distance cutoff to any of the cluster isolates. To construct the next cluster, an isolate was randomly chosen from the un-clustered list of isolates, and the same algorithm was repeated. The construction of clusters ended when the continuously updated list of un-clustered isolates contained no remaining isolates. For each geographic region we found that most (>80%) human isolates formed a single cluster using the distance cutoff of 4% of total variance.

We then identified all the avian isolates that fall within a distance corresponding to 2% variation to all the human isolates in the identified cluster (Figure S1 shows a schematic representation of this method). This subset of the avian isolates was then used to identify the set of avian isolates closest to the human isolates. PCA plots showing human and closely clustering avian isolates are shown in Figure 2.

### Detection of residues in the human isolates with significant amino acid frequency differences from the avian isolates

For isolates from each region and year, we computed the significance of differences in amino acid frequencies at each residue between the human and avian isolates. We treat amino acids at a residue in human isolates from a given year and region as samples drawn from the distribution of amino acids present at the same residue in the avian isolates of the same year. We then used the multinomial formula for sampling to evaluate the likelihood of sampling the human amino acid configuration from the amino acid distribution from the avian isolates. At a given residue in isolates from a given year and region, let 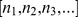 be the observed counts for amino-acids 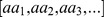 in the human isolates, and let 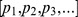 be the corresponding amino-acid frequencies in the avian isolates. The likelihood of seeing these counts in human isolates, given that they are sampled randomly from the avian isolates, is given by
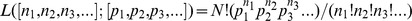
where 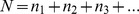 is the total number of human isolates from a given year and region.

An empirical p-value for this likelihood was computed by drawing 10^8^ random sample sets of equal size to the human isolates from the distribution of amino-acid residues in avian isolates, and counting the fraction of such realizations with a lower likelihood than observed. To correct for population structure in H5N1 isolates from each geographic region, we performed this analysis only on the subset of avian isolates that clustered closest to human isolates, as described in the previous section.

### Detection of residues in human isolates with low probability of evolving neutrally from the avian H5N1 isolates

We adapted the method introduced by Pan and Deem [18] to compute the probabilities of evolving the observed amino-acid configurations at each site of human isolates from the avian isolates of the previous year. If the amino-acid frequencies in the avian isolates for the previous year (say *y-1*) were observed to be 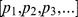, then the theoretically evolved frequencies 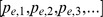 can be computed using the protein evolution model 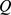 as
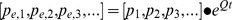
where 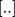 is a row vector, 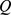 is a 20*20 matrix, and 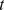 is measured in units of mutation rate, which we assume to be the substitution rate of 4.77*10^−3^/site/year [27]. For 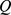, we used an Influenza specific protein evolution model FLU, as calculated by Dang et al [19]. Using these evolved frequencies, we compute the likelihood of observing the amino-acid configuration of human isolates of year *y* as before:
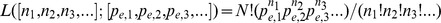



To compute the significance (p-value) of this likelihood value, we randomly generated 10^8^ samples from the evolved distribution 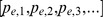 and calculated the likelihood using the above formula for each of these samples. The empirical p-value of the observed likelihood value is the fraction of these 10^8^ samples with lower likelihoods than the one observed.

### Sensitivity to ascertainment bias

The database contained far fewer human isolates than avian isolates, with some years having very few (sometimes only 10) human isolates. In such cases, a few outlier samples could bias the results. To correct for this, we studied the sensitivity of our results when only a subset of the full dataset is used. We randomly chose 1,000 subsets containing 75% of human and 1,000 subsets of 75% of avian isolates in each year. Our analysis was repeated on the 1,000*1,000 = 10^6^ combinations of these subsets of human and avian isolates. We then calculated the mean and standard deviations for the likelihoods of amino acid frequency difference and likelihood of neutral evolution using the methods described above for all the combinations. In our final results we only retained those residues which either had a mean likelihood of amino acid frequency difference <10^−5^ and neutral evolution likelihood <10^−3^ or vice versa.

### Sensitivity to mutation rate variation

Because the computation of probabilities of neutral evolution of human isolates from the avian viral isolates uses mutation rate as an input parameter, we studied the sensitivity to local variation in mutation rates. We first used the program PhyML [28] to generate the maximum likelihood values for the 4 mutation rate classes of a discrete Γ4 model of variable mutation rates. We used both human and avian isolates from Egypt to be analyzed using PhyML using the model FLU and other default parameters. We obtained the maximum likelihood values for 4 classes of the discrete Γ4 model to be {0.0288, 0.2353, 0.8012, 2.9346}, which we multiplied with the mean rate of 4.77*10^−3^/site/year [28] to get the 4 classes of mutation rates. We then calculated the likelihood of neutral evolution for all the significant sites using each of these rates, and found that all the significant residues had mean likelihood <10^−5^.

## Supporting Information

Figure S1 **Schematic of clustering algorithm in PCA space.** The figure shows the first two principal components PCA of HA amino acid sequences from avian and human isolates from Egypt. A) A sphere of proximity with radius corresponding 1% of the total variance was constructed around each human isolate, and all human isolates connected by overlapping circles were clustered together. Avian isolates located in the same cluster as human isolates were added to the cluster. B) Results from the implementation of the algorithm using the first 4 principal components and spheres of proximity with radius corresponding to 2% of the total variance.
(PNG)

Figure S2 **Location of significant residues on H5 Hemagglutinin.** Loci on the H5 hemagglutinin protein which show transmission bias. Structure data was obtained from PDB file 2IBX [15], and was analyzed using the program PyMol. Red, blue and purple spheres represent loci under transmission bias in Indonesia, Egypt and China respectively.
(PNG)

Figure S3 **Loci with significantly different frequencies after correcting for biased transmission of H5N1.** A) The I-513 mutation in HA from H5N1 strains circulating in Egypt shows enrichment in human isolates in years 2007 and 2008 compared to avian isolates. B) The N-183 mutation in HA from H5N1 strains circulating in Indonesia shows enrichment in human isolates compared to avian isolates. The numbers in parentheses are the number of human isolates in each region and year.
(PNG)

Table S1 Table S1A, Closely clustering avian and human isolates (methods) identified using HA sequences from H5N1 strains in Egypt; Table S1B, Closely clustering avian and human isolates (methods) identified using HA sequences from H5N1 strains in China; Table S1C, Closely clustering avian and human isolates (methods) using HA sequences from H5N1 strains in Indonesia.
(ZIP)

Table S2 **Residues in human isolates with significantly different amino acid frequencies and significantly low probability of neutral evolution from the entire avian viral pool.** The probability of neutral evolution and significance of amino acid frequency difference at the identified residues are listed when human isolates are compared with all avian isolates and with the subset of closely clustering avian isolates (Methods). Information on location of residues in functional regions of HA was used from Duvvuuri et al [11]. Amino-acid frequencies for these residues are shown in Table 1 and Table S3.
(XLS)

Table S3 **Transmission bias mutations which are at intermediate frequency in closely clustering human and avian isolates.** Residues with intermediate frequency major amino acids in closely clustering human isolates which have significantly low probability to neutrally evolve from and significant differences in amino-acid frequencies from the entire avian viral pool of each geographical region and year. The numbers in the rows next to each country's names indicate the number of samples in each class, and the years these were first reported in each country.
(XLS)

Table S4 **Residues in human isolates with significant amino-acid frequency difference from and significantly low probability to evolve from avian isolates after correcting for transmission bias of H5N1 strains from birds to humans.** To correct for bird-to-human transmission bias of H5N1 strains, human isolates were compared with closely clustering avian isolates (Methods) in each geographic region. Significance cutoffs were set at the multiple hypothesis threshold 1/(572*3*5) for either neutral evolution from the previous year or significantly different amino acid frequency, so long as the other p-value<0.01. These residues were robust to jack-knifing analysis (Methods). Information about location of residues in functional regions was used from Duvvuri et al [11].
(XLS)
