# Supplementary figures and images for: Bird to Human Transmission Biases and Vaccine Escape Mutants in H5N1 Infections
Source: PLoS One. 2014 Jul 2;9(7):e100754. doi: 10.1371/journal.pone.0100754 (PMC4079711; doi:10.1371/journal.pone.0100754)

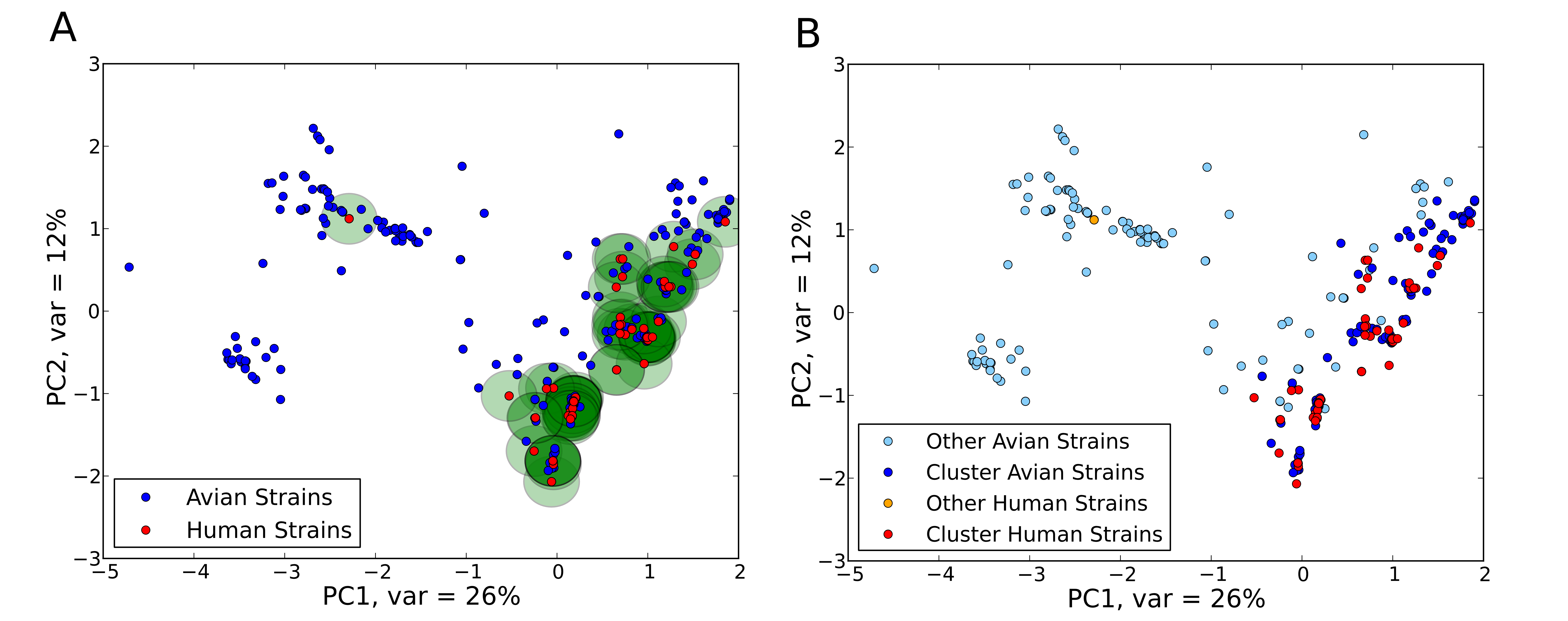

Supplement: Figure S1 — Schematic of clustering algorithm in PCA space. The figure shows the first two principal components PCA of HA amino acid sequences from avian and human isolates from Egypt. A) A sphere of proximity with radius corresponding 1% of the total variance was constructed around each human isolate, and all human isolates connected by overlapping circles were clustered together. Avian isolates located in the same cluster as human isolates were added to the cluster. B) Results from the implementation of the algorithm using the first 4 principal components and spheres of proximity with radius corresponding to 2% of the total variance. (PNG) [file pone.0100754.s001.png]

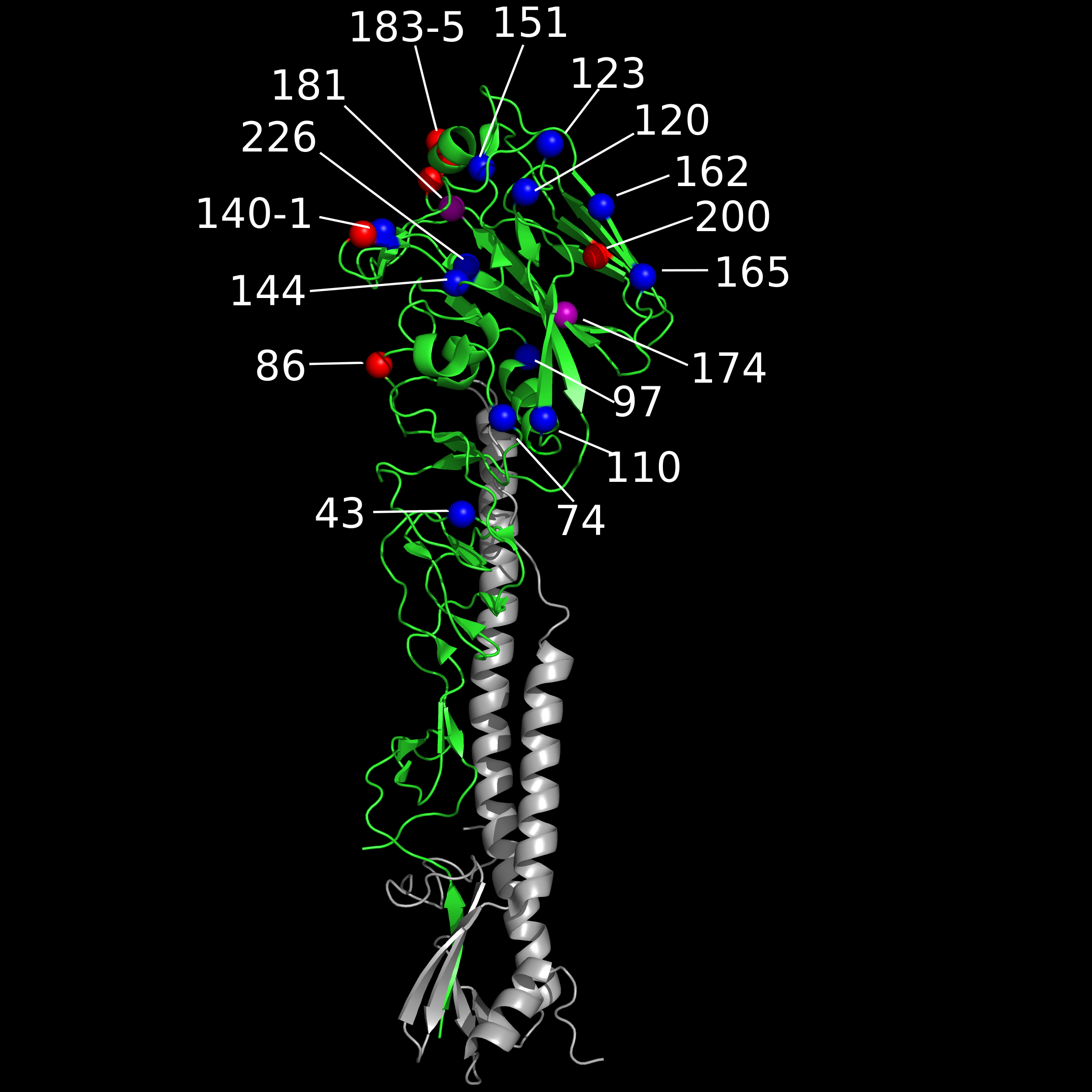

Supplement: Figure S2 — Location of significant residues on H5 Hemagglutinin. Loci on the H5 hemagglutinin protein which show transmission bias. Structure data was obtained from PDB file 2IBX [15], and was analyzed using the program PyMol. Red, blue and purple spheres represent loci under transmission bias in Indonesia, Egypt and China respectively. (PNG) [file pone.0100754.s002.png]

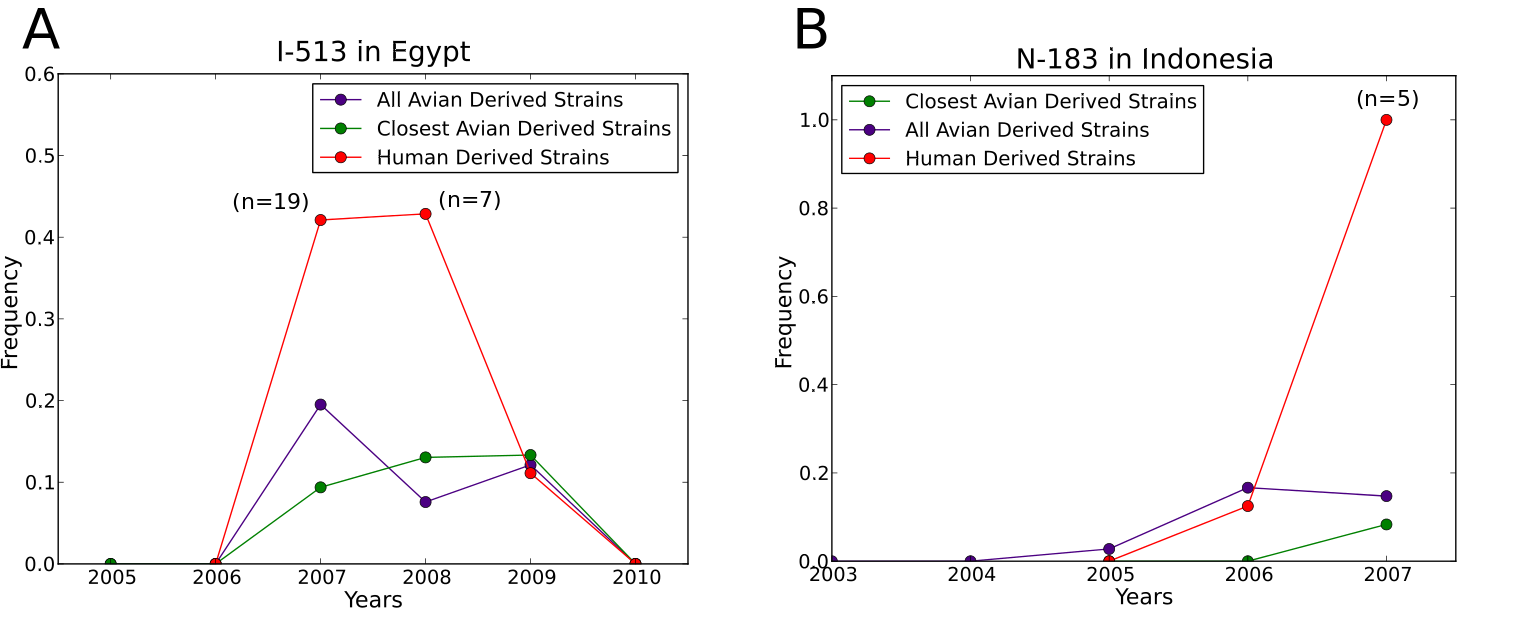

Supplement: Figure S3 — Loci with significantly different frequencies after correcting for biased transmission of H5N1. A) The I-513 mutation in HA from H5N1 strains circulating in Egypt shows enrichment in human isolates in years 2007 and 2008 compared to avian isolates. B) The N-183 mutation in HA from H5N1 strains circulating in Indonesia shows enrichment in human isolates compared to avian isolates. The numbers in parentheses are the number of human isolates in each region and year. (PNG) [file pone.0100754.s003.png]
